# Coexisting benign and malignant tumours: a cardiac angiosarcoma case report

**DOI:** 10.1093/ehjcr/ytag591

**Published:** 2026-08-18

**Authors:** Tiemen E T Holtrop, Sulayman el Mathari, Jonathan Etnel, Loudy P Priesterbach-Ackley, Jolanda Kluin

**Affiliations:** Department of Cardiothoracic Surgery, Erasmus MC Rotterdam, Dr. Molewaterplein 40, Rotterdam 3015GD, The Netherlands; Department of Cardiothoracic Surgery, Erasmus MC Rotterdam, Dr. Molewaterplein 40, Rotterdam 3015GD, The Netherlands; Department of Cardiothoracic Surgery, Erasmus MC Rotterdam, Dr. Molewaterplein 40, Rotterdam 3015GD, The Netherlands; Department of Pathology, Erasmus MC Rotterdam, Dr. Molewaterplein 40, Rotterdam 3015GD, The Netherlands; Department of Cardiothoracic Surgery, Erasmus MC Rotterdam, Dr. Molewaterplein 40, Rotterdam 3015GD, The Netherlands

**Keywords:** Cardiac angiosarcoma, Haemorrhagic pericardial effusion, Diagnostic pitfall, Case report

## Abstract

**Background:**

Primary cardiac tumours are rare. When patients present with both cardiac and extracardiac masses, clinicians may opt to biopsy accessible peripheral lesions to guide diagnosis, but benign findings can provide false reassurance. This case demonstrates the diagnostic pitfall of this approach when multiple concurrent pathologies coexist.

**Case summary:**

A 79-year-old male presented with progressive peripheral oedema, dyspnoea, and chest pain. Echocardiography revealed a 6.5 cm mobile intracardiac mass originating from the right atrial appendage. Pericardiocentesis drained 1100 cc of haemorrhagic fluid with negative cytology. PET-CT showed FDG uptake in the cardiac mass as well as multiple extracardiac lesions. Biopsy of a left upper arm lesion revealed a benign peripheral nerve sheath tumour. Photon-counting CT deemed angiosarcoma less likely due to limited contrast enhancement. These findings combined initially lowered suspicion for cardiac malignancy. The patient underwent surgical resection via median sternotomy. Histopathology revealed a high-grade angiosarcoma with positive resection margins (CD31+, CD34+, ETS-Related Gene [ERG]+, Ki-67 40%–60%). The postoperative course was uncomplicated, and the patient was transferred to the referring hospital on postoperative day four.

**Discussion:**

This case demonstrates that benign pathology from peripheral lesions should not eliminate suspicion for cardiac malignancy when clinical features such as haemorrhagic pericardial effusion, right heart failure, and thromboembolic complications are present. Multiple concurrent pathologies can coexist, underlining the limitations of extrapolating peripheral biopsy results to cardiac masses.

Learning points

- Benign pathology from extracardiac lesions should not be extrapolated to cardiac masses when clinical features suggest malignancy.
- Negative pericardial effusion cytology does not exclude cardiac angiosarcoma when other clinical red flags for malignancy are present.

## Introduction

Angiosarcomas are the most common primary cardiac malignancy in adults, accounting for approximately 20%–40% of all cardiac sarcomas.^1,2^ These aggressive tumours arise predominantly from the right atrium (RA) and typically present with non-specific symptoms including dyspnoea, chest pain, pericardial effusion, and signs of right heart failure,^1,3^ often leading to delayed diagnosis. Despite multimodal treatment, prognosis remains poor with a median survival of 14–17 months.^2,4^

Multiple advanced imaging modalities exist in the diagnostic approach to cardiac masses, including positron emission tomography–computed tomography (PET-CT) and cardiac magnetic resonance imaging (CMR), yet tissue diagnosis remains crucial.^5^ When patients present with both cardiac and extracardiac lesions, biopsy of a more accessible peripheral lesion may influence diagnosis, but this approach, while often more practical, could lead to misdiagnosis when multiple pathologies coexist.

We present a case of a 79-year-old man with a large RA mass and multiple extracardiac lesions. A benign peripheral nerve sheath tumour from a left upper arm biopsy, combined with limited contrast enhancement on photon-counting CT (PCCT), initially lowered suspicion for cardiac malignancy. Surgical resection revealed a high-grade angiosarcoma with positive margins. We demonstrate the diagnostic pitfalls of extrapolating extracardiac lesion pathology to cardiac masses.

## Summary figure

**Figure:**
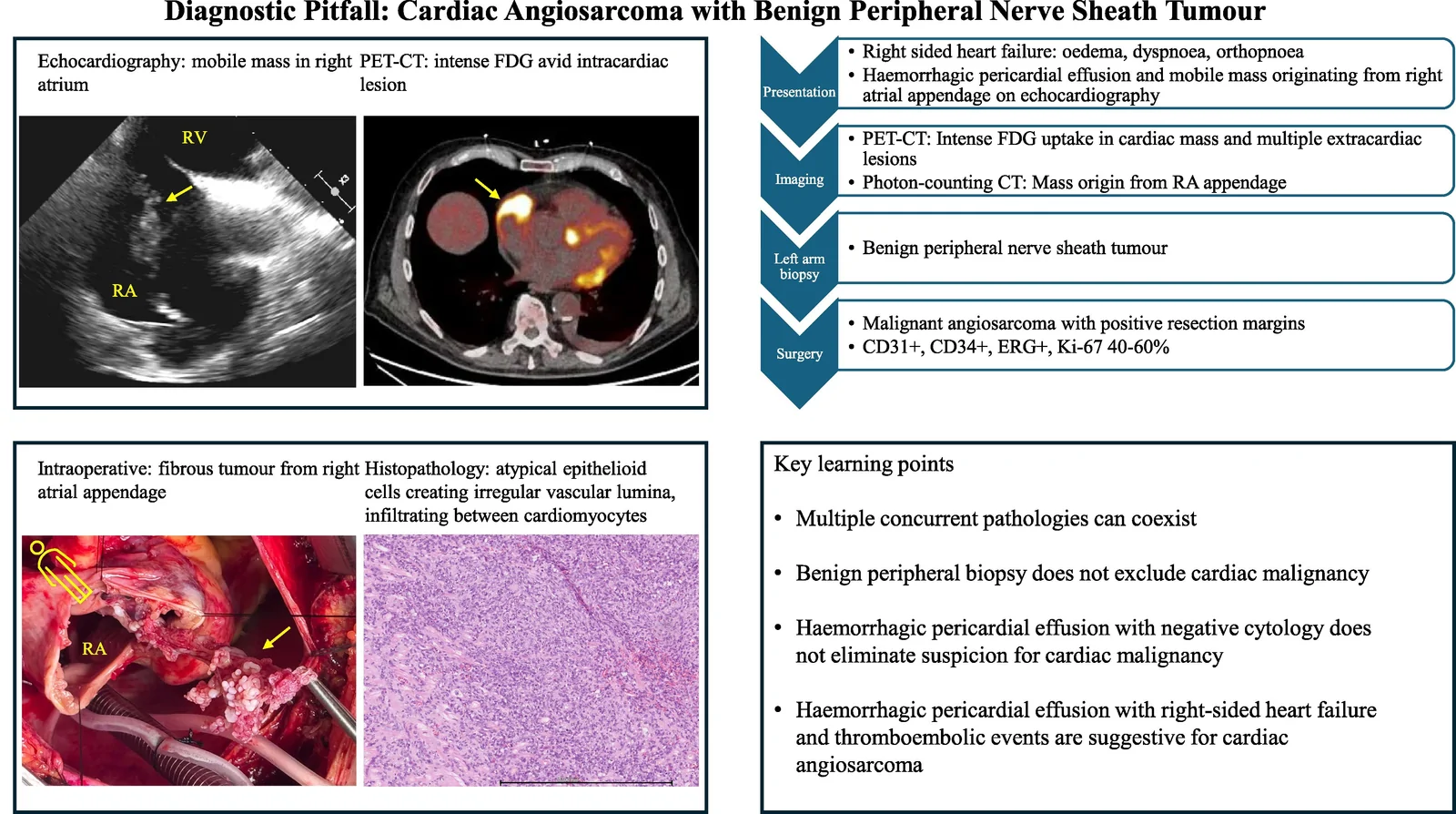


## Case presentation

**Figure 1:**
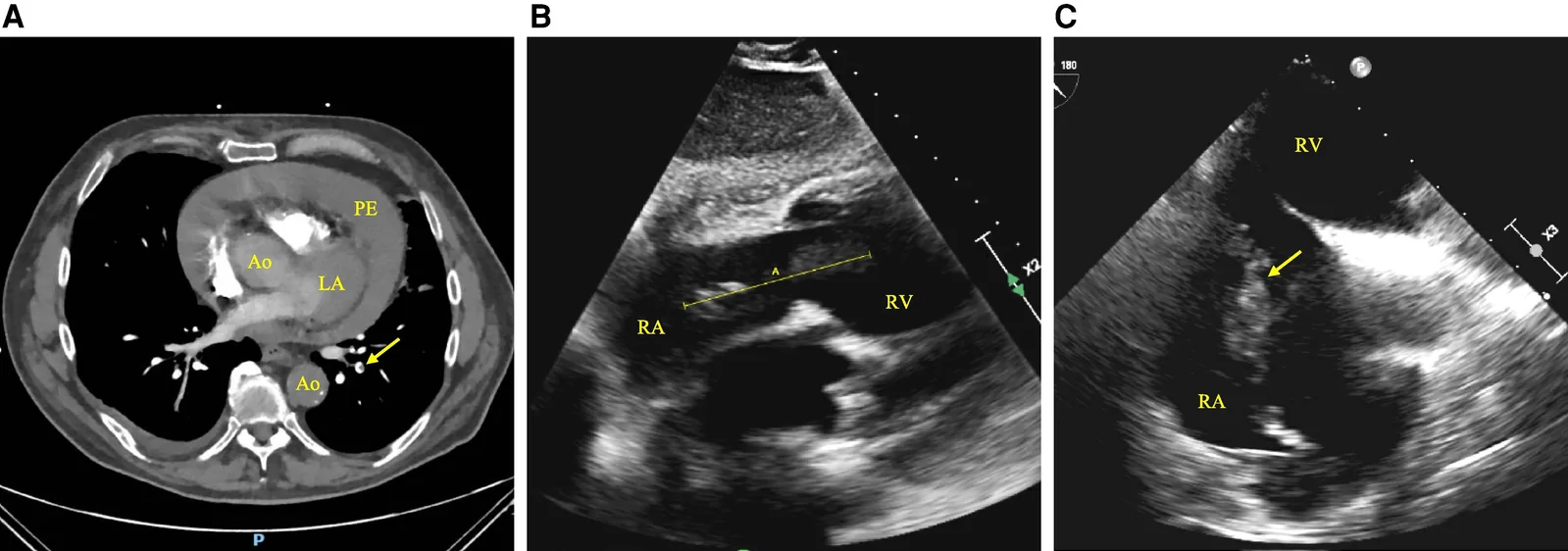
Imaging at first presentation at referring hospital. (*A*) Computed tomography demonstrating large pericardial effusion and segmental pulmonary embolisms (arrow). (*B*) Transthoracic echocardiogram showing a large mobile mass (6.5 cm) in the right atrium. (*C*) Transoesophageal echocardiogram showing a pedunculated mass originating from the right atrial appendage (arrow). Ao, aorta; LA, left atrium; PE, pericardial effusion; RA, right atrium; RV, right ventricle.

A 79-year-old male presented with a two-week history of progressive peripheral oedema, exertional dyspnoea, orthopnoea, and intermittent chest pain. Physical examination revealed pitting oedema up to the waist. CT imaging showed bilateral segmental pulmonary embolisms and large pericardial effusion (*Figure 1A*). Transthoracic and transoesophageal echocardiography demonstrated a large pedunculated mobile mass (approximately 6.5 cm) originating from the RA appendage, possibly extending through the tricuspid valve (*Figure 1B, C*). Pericardiocentesis drained 1100 cc of haemorrhagic fluid, with negative cytology for malignancy. Diuretics were started for volume overload.

**Figure 2:**
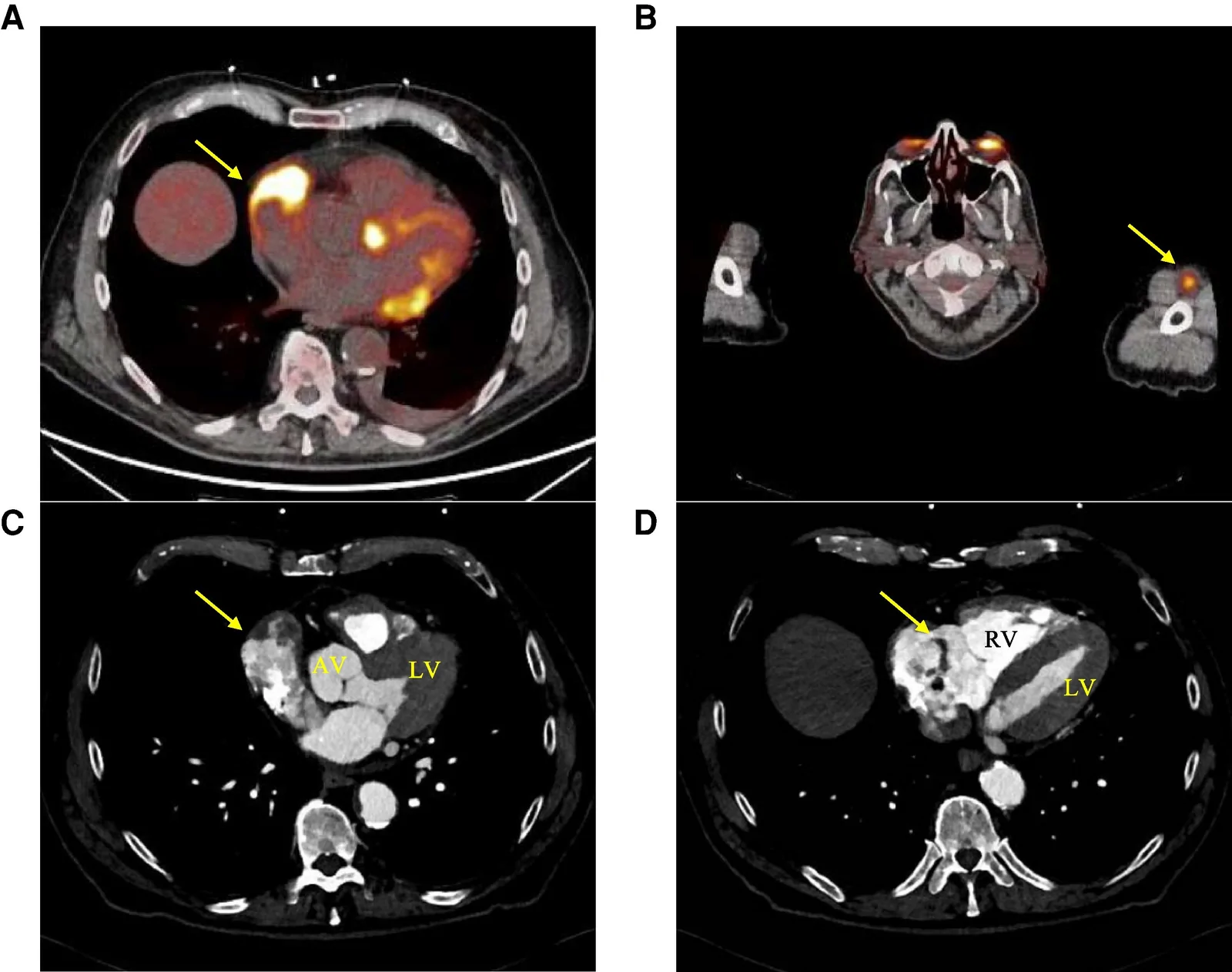
Advanced imaging at tertiary centre. (*A*) Positron emission tomography-computed tomography (PET-CT) demonstrating intense FDG uptake in the cardiac mass (arrow). (*B*) PET-CT showing an FDG-avid lesion in the left upper arm. (*C*) Photon-counting CT confirming mass origin from the right atrial appendage base with diffuse myocardial thickening (arrow). (*D*) Photon-counting CT demonstrating linear hypodense structure extending medially and dorsally from the right atrial appendage towards the inferior vena cava (arrow). AV, aortic valve; LV, left ventricle; RV, right ventricle.

**Figure 3:**
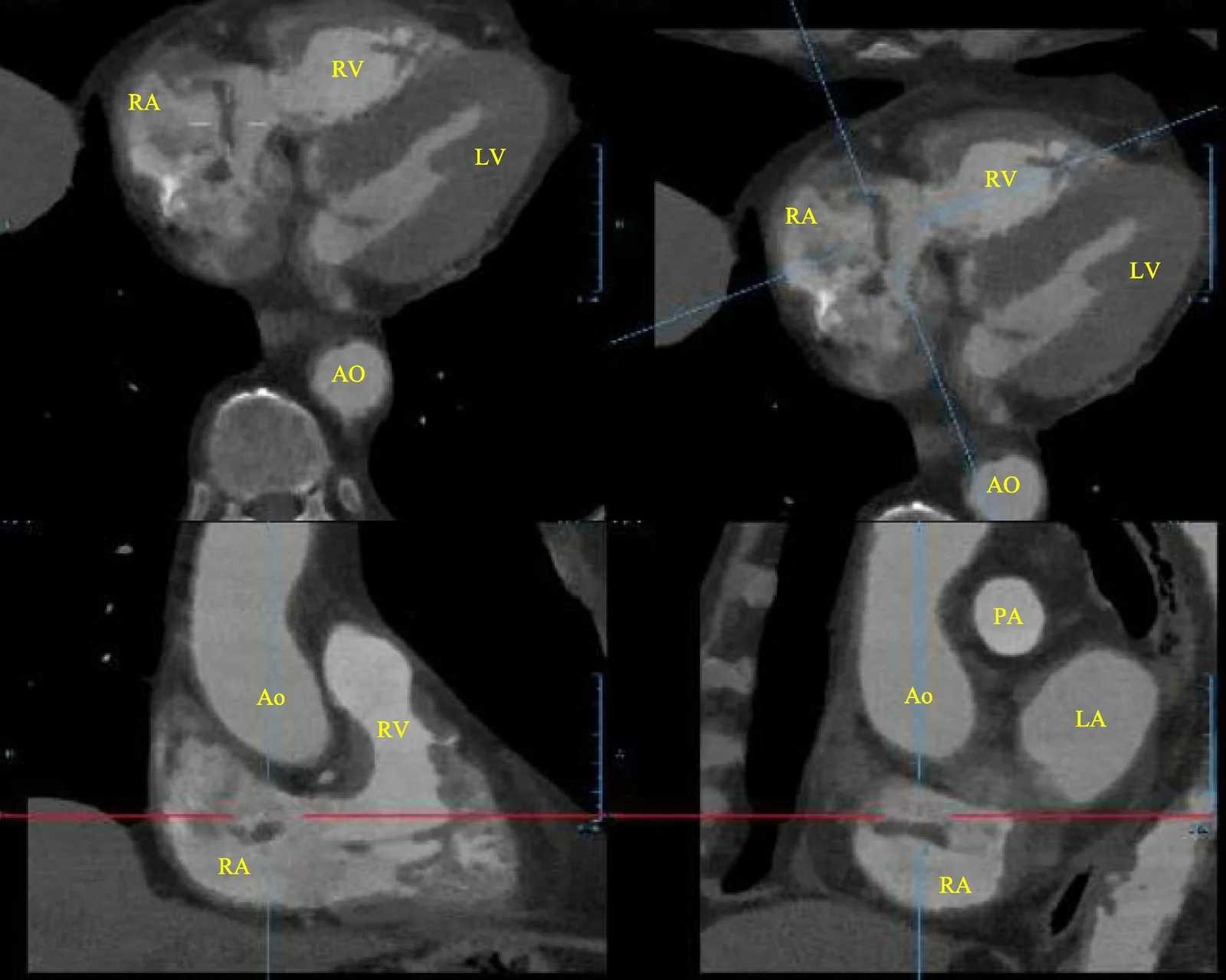
Photon-counting computed tomography multiplanar reconstruction demonstrating a mobile linear hypodense structure (measuring 39.8 mm) extending from the right atrial appendage base medially and dorsally towards the inferior vena cava, with diffuse irregular thickening of the right atrial appendage myocardium. Ao, aorta; LA, left atrium; LV, left ventricle; PA, pulmonary artery; RA, right atrium; RV, right ventricle.

The patient was transferred to a tertiary centre, where PET-CT showed intense FDG uptake in the cardiac mass and multiple FDG-avid nodular lesions, including the left upper arm, right shoulder, and intercostal space (*Figure 2A, B*). Image-guided biopsy of the left upper arm lesion revealed a benign peripheral nerve sheath tumour. CMR imaging was not performed, as the highly mobile nature of the mass could limit tissue characterization due to motion artefacts, and the thromboembolic risk necessitated rapid diagnostic evaluation. Instead, PCCT confirmed the mass originating from the RA appendage base with diffuse thickening (*Figure 2C, D* and *Figure 3*). While thrombus was initially considered the primary diagnosis, malignancy could not be excluded given the FDG-avid uptake. PCCT showed limited contrast enhancement of the mass (40 HU pre-contrast, 81 HU post-contrast, with the mass being poorly demarcated on the non-contrast phase). Angiosarcoma was deemed less likely, ultimately lowering suspicion for cardiac malignancy.

**Figure 4:**
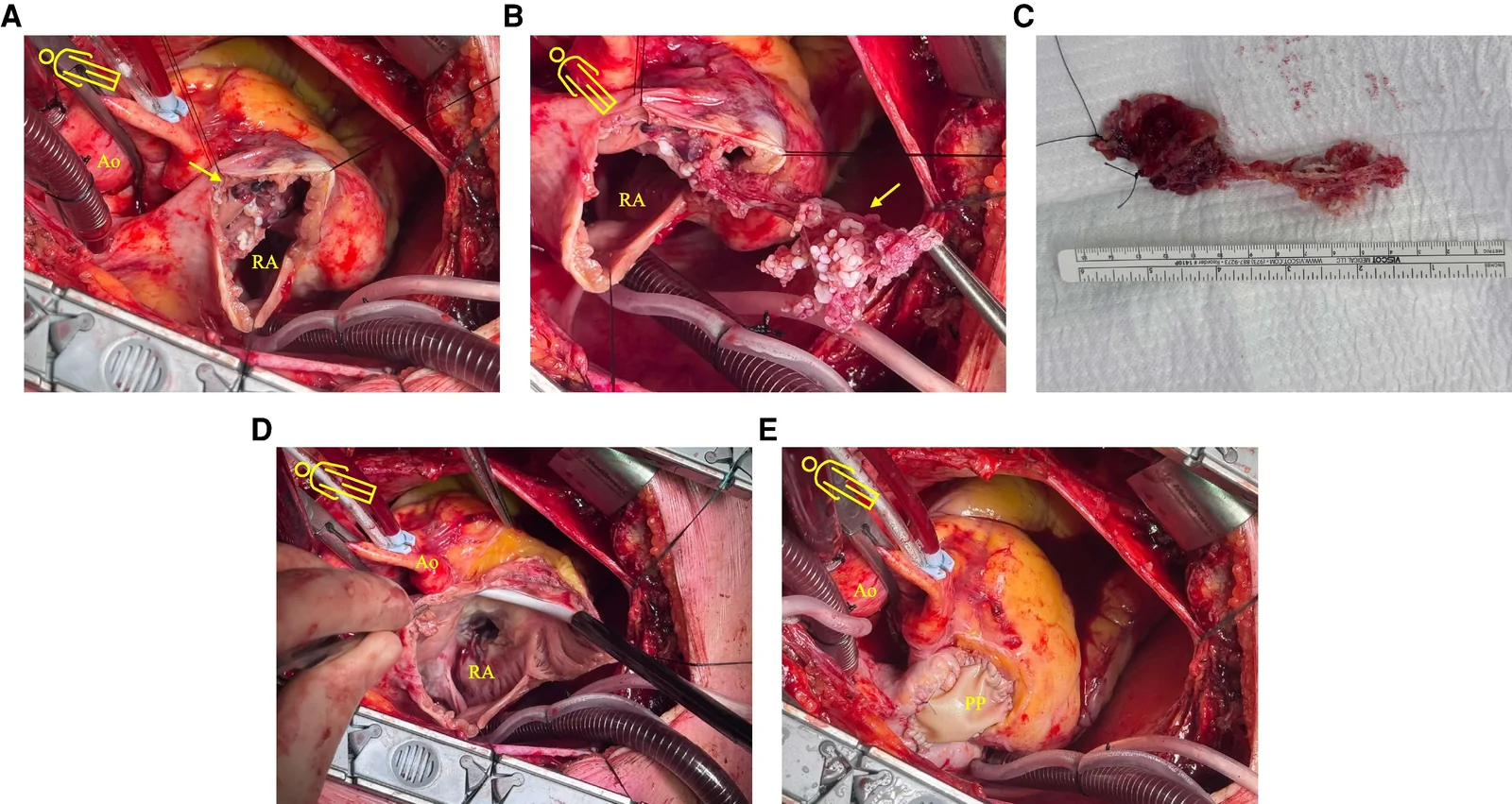
Intraoperative findings. (*A*) Initial surgical view showing opened right atrium with tumour base (arrow). (*B*) Specimen held by forceps demonstrating large fibrous tumour with nodular protrusions and broad-based attachment (arrow). (*C*) Excised tumour specimen with ruler demonstrating size. (*D*) Cryoablation of residual tumour tissue near the right coronary artery. (*E*) Right atrial reconstruction with bovine pericardial patch. Ao, aorta; PP, pericardial patch; RA, right atrium.

Surgical resection was indicated to prevent luxation and embolic events. The patient underwent median sternotomy with cardiopulmonary bypass. Intraoperatively, a large fibrous tumour with nodular protrusions was identified, with a broad attachment base invasively extending from the RA appendage apex into the anterior atrioventricular groove (*Figure 4A–C*). The tumour was resected with full-thickness resection of the involved RA wall. A small rim of affected right atrial tissue was left *in situ* due to involvement of the right coronary artery and the atrioventricular junction. This area was treated with cryoablation (*Figure 4D*). The resulting defect in the right atrial wall was reconstructed using a bovine pericardial patch (*Figure 4E*).

The postoperative course was uncomplicated, and the patient was transferred to the referring hospital in stable condition on postoperative day four.

**Figure 5:**
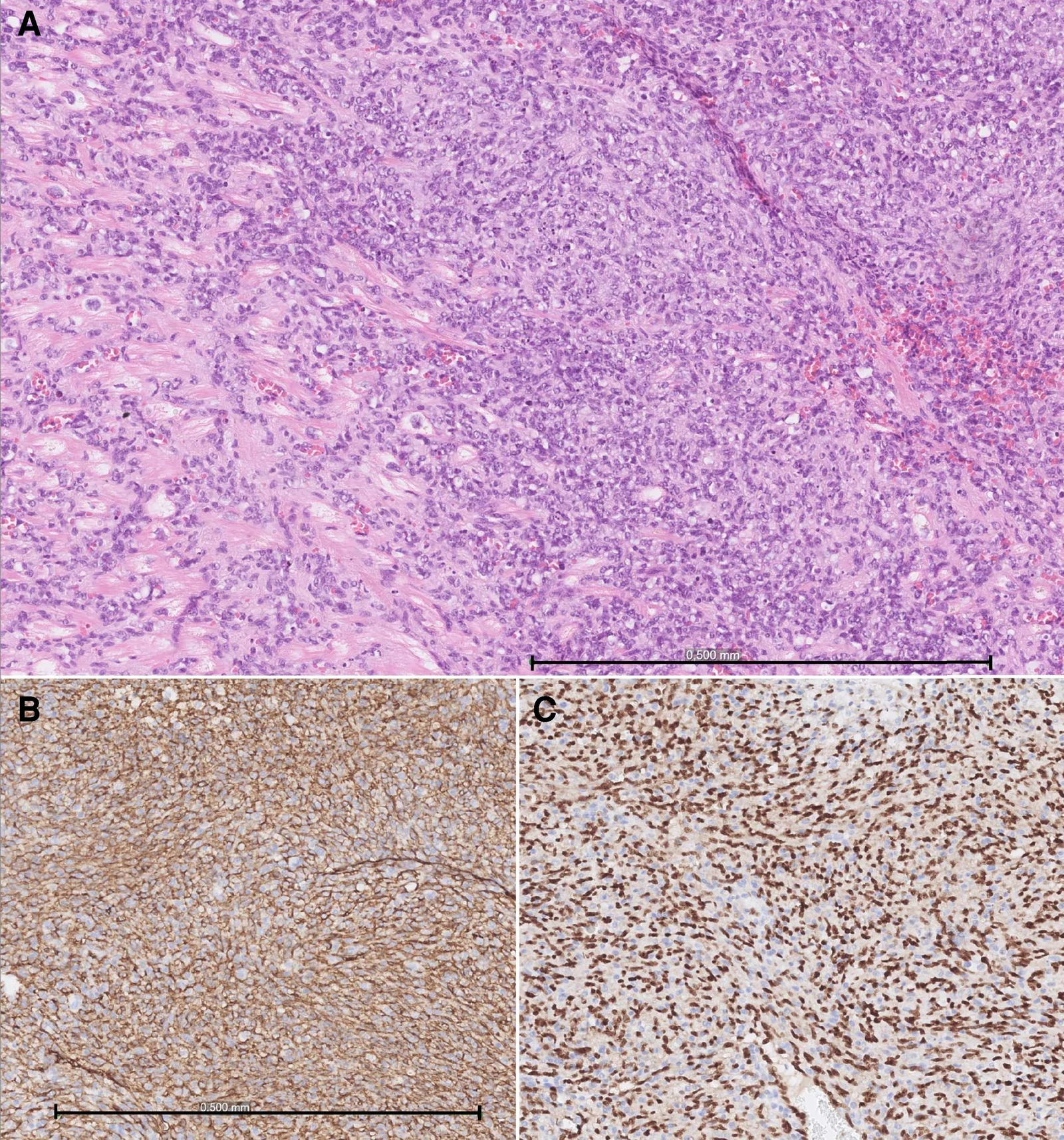
Histopathological findings. (*A*) Haematoxylin and eosin stain of the cardiac lesion (20 × magnification), showing atypical epithelioid cells creating irregular vascular lumina filled with erythrocytes, and infiltrating between cardiomyocytes. (*B*) Positive CD34 immunohistochemistry staining of lesional cells (20 × magnification). (*C*) Positive ERG immunohistochemistry staining of lesional cells (20 × magnification).

Histopathological examination (*Figure 5*) revealed papillary structures and sheets of atypical oval to epithelioid cells with numerous mitotic figures and extensive invasive growth between cardiomyocytes and infiltrating in epicardial fat, with positive resection margins. Some areas showed irregular slit-like vascular lumina containing erythrocytes, rather than well-formed vessels. Immunohistochemistry demonstrated positive staining of the lesional cells for CD31, CD34, and ETS-Related Gene (ERG). The Ki-67 proliferation index was high, ranging from 40–60%. Pankeratin was negative, Calretinin, S100, and SOX10 were predominantly negative, and there was no loss of INI1 or H3K27me3. The final diagnosis was high-grade angiosarcoma with positive resection margins extending to both the apex and atrioventricular groove.

Following discharge, the case was discussed in a multidisciplinary sarcoma meeting, and referral for adjuvant radiotherapy was recommended given the positive resection margins. The patient subsequently underwent five well-tolerated sessions of stereotactic radiotherapy and remained free of cardiac symptoms, with ongoing surveillance by CMR and thoracic CT.

## Discussion

This case highlights a diagnostic reasoning pitfall in the evaluation of cardiac masses when multiple extracardiac lesions are present. While biopsy of accessible peripheral lesions can be pragmatic, benign findings can lower suspicion for cardiac malignancy when multiple concurrent pathologies coexist. In this case, the benign peripheral nerve sheath tumour, combined with limited contrast enhancement on PCCT, initially lowered suspicion for cardiac malignancy, despite clinical red flags including haemorrhagic pericardial effusion, right heart failure, and thromboembolic complications. While surgical resection was indicated regardless of the preoperative diagnosis due to the size, risk of thromboembolic events, and luxation of the mass, the preoperative assessment underestimated the likelihood of malignancy. Surgical resection ultimately revealed a high-grade angiosarcoma with extensive invasive growth, emphasizing the limitations of extrapolating pathology from anatomically distant lesions when diagnosing cardiac masses. This case therefore illustrates the diagnostic challenge in weighing conflicting findings and highlights the need to maintain clinical suspicion when features suggestive of malignancy are present.

Our patient’s clinical presentation, combining right heart failure symptoms, haemorrhagic pericardial effusion, and thromboembolic complications such as pulmonary embolisms,^6^ corresponds with the typical complaints of cardiac angiosarcomas^3^ and should have conferred a high pre-test probability of malignancy, independent of the imaging and peripheral biopsy findings. Haemorrhagic pericardial effusion is often present in angiosarcomas,^7^ though pericardial fluid cytology has low sensitivity for diagnosis.^8,9^ The presence of multiple FDG-avid lesions on PET-CT added diagnostic complexity, as benign nerve sheath tumours, angiosarcomas, and other cardiac malignancies can all demonstrate metabolic activity.^10,11^ Therefore, imaging alone cannot exclude malignancy. In this case, the limited contrast enhancement on PCCT was initially interpreted as arguing against angiosarcoma, given the typically vascularized nature of these tumours.^3^ However, cardiac angiosarcomas may in fact demonstrate heterogeneous or limited contrast enhancement, particularly in areas of necrosis or haemorrhage, which can complicate imaging interpretation. This represents an important imaging pitfall and highlights the limitations of imaging-based exclusion of malignancy, underscoring the need to weigh imaging findings against the broader clinical picture. While CMR can be valuable for tissue characterization of cardiac masses, including late gadolinium enhancement and T1-weighted imaging in angiosarcomas, it was not performed in this case. The highly mobile nature of the mass would have limited reliable tissue characterization due to motion artefacts, while the thromboembolic risk necessitated rapid evaluation. In retrospect, CMR might have raised earlier suspicion for malignancy, although its added value in highly mobile cardiac masses remains limited. These findings underscore that tissue diagnosis from the primary cardiac lesion remains the most reliable method for diagnosis. Endomyocardial biopsy (EMB) can provide clarity in complex cases,^12,13^ although its value depends on the anticipated impact on management. In our patient, EMB was not pursued as surgical resection was already indicated. While tissue diagnosis remains the gold standard for cardiac masses, direct cardiac tissue sampling through EMB carries inherent procedural risks such as bleeding and arrhythmias.^12,13^ Complete surgical resection remains the cornerstone of treatment and an important prognostic factor,^4^ as patients with unresected disease have a mean survival as low as 3.8 months.^14^ In our case, invasive growth and proximity to the right coronary artery resulted in positive resection margins. Although the contradictory findings did not alter the treatment course, false reassurance may have influenced perioperative counselling.

In the presence of additional extracardiac lesions, biopsy of a more accessible lesion may be a pragmatic alternative, particularly when a known primary malignancy or typical metastatic pattern makes pathological concordance probable. However, when primary cardiac malignancy is considered, particularly in the presence of concordant clinical features, direct tissue sampling of the cardiac lesion remains most reliable. Although such considerations cannot be formalized in a single algorithm, this case underscores that the choice between peripheral biopsy and direct cardiac tissue sampling should be informed by both pre-test probability of primary cardiac malignancy and the clinical context. Primary cardiac tumours are exceptionally rare,^3^ and the procedural risk of cardiac tissue sampling often favours biopsy of more accessible lesions, although benign findings from such biopsies should not eliminate suspicion of cardiac malignancy when clinical features suggest otherwise.

## Data Availability

No new data were generated or analysed in support of this research.

## Lead author biography

Tiemen Holtrop, MD, completed his medical degree at Leiden University Medical Center, the Netherlands. He completed a research fellowship at Massachusetts General Hospital in Boston, where he focused on trauma surgery outcomes and pain management strategies in patients with rib fractures. He is currently an ANIOS (junior resident) in Cardiothoracic Surgery at Erasmus Medical Center in Rotterdam. His research interests include cardiothoracic surgery, surgical oncology, and trauma care, with emphasis on diagnostic challenges.

## Notes

**Consent:** The authors confirm that written informed consent for submission and publication of this case report, including the images and associated text, has been obtained from the patient in line with Committee on Publication Ethics (COPE) guidance.
